# Proton Pumping and Non-Pumping Terminal Respiratory Oxidases: Active Sites Intermediates of These Molecular Machines and Their Derivatives

**DOI:** 10.3390/ijms221910852

**Published:** 2021-10-07

**Authors:** Sergey A. Siletsky, Vitaliy B. Borisov

**Affiliations:** 1 Department of Bioenergetics, Belozersky Institute of Physico-Chemical Biology, Lomonosov Moscow State University, Leninskie Gory, Moscow 119991, Russia; 2 Department of Molecular Energetics of Microorganisms, Belozersky Institute of Physico-Chemical Biology, Lomonosov Moscow State University, Leninskie Gory, Moscow 119991, Russia; bor@belozersky.msu.ru

**Keywords:** membrane proteins, terminal oxidases, cytochrome oxidase, cytochromes, proton pump, electrogenic mechanisms, reactive oxygen species

## Abstract

Terminal respiratory oxidases are highly efficient molecular machines. These most important bioenergetic membrane enzymes transform the energy of chemical bonds released during the transfer of electrons along the respiratory chains of eukaryotes and prokaryotes from cytochromes or quinols to molecular oxygen into a transmembrane proton gradient. They participate in regulatory cascades and physiological anti-stress reactions in multicellular organisms. They also allow microorganisms to adapt to low-oxygen conditions, survive in chemically aggressive environments and acquire antibiotic resistance. To date, three-dimensional structures with atomic resolution of members of all major groups of terminal respiratory oxidases, heme-copper oxidases, and *bd*-type cytochromes, have been obtained. These groups of enzymes have different origins and a wide range of functional significance in cells. At the same time, all of them are united by a catalytic reaction of four-electron reduction in oxygen into water which proceeds without the formation and release of potentially dangerous ROS from active sites. The review analyzes recent structural and functional studies of oxygen reduction intermediates in the active sites of terminal respiratory oxidases, the features of catalytic cycles, and the properties of the active sites of these enzymes.

## 1. Introduction: General Properties of Terminal Respiratory Oxidases

The membrane-embedded terminal respiratory oxidases include two main groups of functionally similar, but structurally and evolutionarily strikingly different superfamilies: heme-copper oxidases and *bd*-type cytochromes. The superfamily of heme-copper oxidases (HCOs) includes cytochrome oxidases (COXs) of mitochondria from higher and lower eukaryotes, HCOs of most aerobic prokaryotes, and structurally related NO reductases [1]. A distinctive feature of these enzymes is the presence of catalytic binuclear center (BNC) consisting of closely located iron ion of the heme group and copper ion (non-heme iron ion in NO reductases) [2]. The *bd*-type cytochrome superfamily members have to date been found only in Bacteria and Archaea [3,4,5,6].

HCOs catalyze the transfer of electrons from quinols or cytochromes to oxygen with the formation of water coupled to the generation of proton motive force [2,7,8,9,10,11,12,13,14,15,16,17,18,19,20,21,22]. In contrast to *bd*-type oxidases, HCOs generate the proton motive force not only by the transfer of electrons and protons to the catalytic center from different sides of the membrane but also due to the unique ability for redox-coupled directed proton pumping through the membrane [23]. NO reductases, which are structurally similar to HCOs, reduce NO to N_2_O and are utilized by a number of pathogenic bacteria for denitrification under microaerobic and anaerobic conditions that occur in many host tissues [24].

The stoichiometric reactions for a number of members of HCO and *bd* oxidases are given below. They differ in the electron donor in the oxygen-reductase reaction, the presence of protons pumped through the membrane, and their number per 1 electron.

(1) *aa_3_*-type heme-copper oxidase from *Rhodobacter sphaeroides* (A family)

4 cyt c^2+^ + 8H^+^_in_ + O_2_ → 4 cyt c^3+^ + 2H_2_O + 4H^+^_out_

(2) *ba_3_*-type heme-copper oxidase from *Thermus thermophilus* (B family)

4 cyt c^2+^ + (4 + n) H^+^_in_ + O_2_ → 4 cyt c^3+^ + 2H_2_O + (n) H^+^_out_

(3) *bo*-type heme-copper oxidase from *Escherichia coli* (A family)

2QH_2_ + 8H^+^_in_ + O_2_ → 2Q + 2H_2_O + 8H^+^_out_

(4) *bd*-type oxidase

2QH_2_ + 4H^+^_in_ + O_2_ → 2Q + 2H_2_O + 4H^+^_out_

Where cyt c is cytochrome *c*; QH_2_ and Q are two-electron-reduced and oxidized forms of quinone (ubiquinone or menaquinone), respectively [12,25]; n~2–4 is the amount of the pumped protons in the B family HCOs [26,27]; H^+^_in_ and H^+^_out_ are the protons taken up from the N phase and released to the P phase, correspondingly.

**Figure 1:**
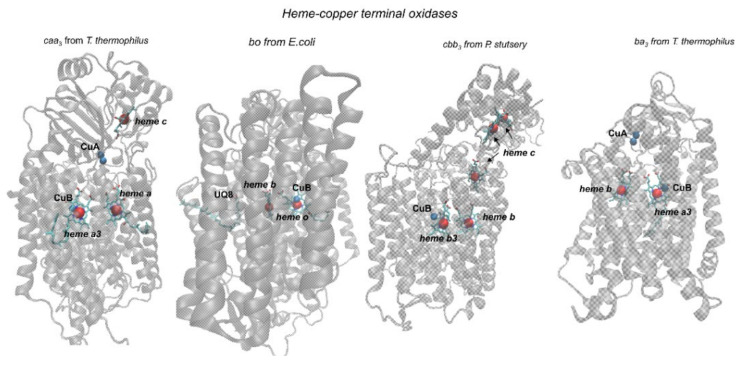
Overall structures of heme-copper cytochrome and quinol oxidoreductases from different families: *caa_3_* from *T. thermophilus* ([28], PDB ID: 2YEV, A2 family), *bo* from *E. coli* ([29], PDB ID: 6WTI, A family), *cbb_3_* from *P. stutzeri* ([30], PDB ID: 5DJQ, C family) and *ba_3_* from *T. thermophilus* ([31], PDB ID: 5NDC, B family). The figure was carried out using Visual Molecular Dynamics software (v1.9.3) [32].

Mammalian mitochondrial cytochrome oxidase contains 13 subunits; the three largest subunits are encoded by the mitochondrial genome. They form the catalytic core of the enzyme and are homologous to the three main subunits found in most typical prokaryotic cytochrome oxidases of the A family. The first subunit contains three redox centers: low-spin heme *a* and oxygen reductase center (BNC) consisting of a closely located iron atom of the high-spin heme *a*_3_ and copper ion (Cu_B_). In prokaryotic HCOs, hemes *a* and *a*_3_ can be replaced with *b-*, *o-*, and *c*-type hemes. The covalently bound *c*-type hemes can also serve as additional redox centers, for example, in *caa*_3_ or *cbb*_3_ oxidases (Figure 1).

A *bd*-type cytochrome consists of two to four subunits [3,33,34,35]. The first subunit of the *bd* oxidase carries a low-spin heme *b*558 that directly accepts electrons from quinol, and a di-heme active site (DHAS) that further receives the electrons from *b*558 [36,37,38,39,40,41,42,43,44,45,46,47,48,49,50]. In contrast to heme-copper oxidases, *bd*-type cytochromes do not contain copper ions. Despite this, they catalyze the oxygen-reductase reaction with very high efficiency. The DHAS of the *bd* enzyme is formed by two high-spin hemes, *d* and *b*595 which are in van der Waals contact [33,34,35]. There are cases when heme *d* is replaced by a *b*-type heme [4,51]. In the case of cytochrome oxidases, the introduction of electrons into the enzyme occurs from the water-soluble cytochrome *c* through the second copper center Cu_A_, consisting of two copper atoms. In the case of heme-copper quinol oxidases, electrons are introduced in the same way as in *bd* oxidases, through a special site of binding and oxidation of quinol (Figure 1). In the case of *caa*_3_ and *cbb*_3_ oxidases, the reduction in Cu_A_ by water-soluble cytochrome *c* is preceded by an intermediate reduction in an additional *c*-type heme redox center(s) (Figure 1). The feature which is used for the classification of HCOs and NO reductases is organization of the intraprotein proton transfer pathways (“channels”) that connect the catalytic center with the membrane cytoplasmic side and ensure the transfer of substrate protons required for water formation, as well as the transfer of protons pumped through the membrane. Based on the structure of these pathways, HCOs are divided into the three main families A, B, and C [52,53,54].

The three-dimensional structure of typical members of the A family has been established by X-ray analysis with a resolution reaching in some cases 1.5 Å. These are COX from bovine heart muscle mitochondria [55,56,57,58], bacterial *aa*_3_-type COX enzymes from *Paracoccus denitrificans* [59] and *R. sphaeroides* [60,61], *bo*_3_-type quinol oxidase from *E. coli* [29,62], cytochrome *caa*_3_ from *T. thermophilus* [28] (Figure 1). Despite the high resolution, the nature of the ligand in the active site of the crystallized enzyme (peroxide, superoxide, chlorine ion, or hydroxyl anion) is still a matter of debate [63]. For some enzymes, the structures of the oxidized and reduced enzyme form, enzyme complex with external ligands CO, CN^–^ [64] and N_3_^–^, the several mutant forms and P and F intermediates [57] of the catalytic cycle have been resolved. Using electron microscopy, the mitochondrial respiratory supercomplex (respirasome) composed of the complexes I, III, and IV (COX) with the CICIII_2_CIV stoichiometry have been resolved [65]. At the same time, two-dimensional electron crystallography has shown that the monomeric form of mammalian COX is functionally active and not an artifact of enzyme purification from the mitochondrial membrane [66]. Despite clarification of the general enzyme topography, a detailed picture of the elementary processes of the transformation of oxygen atoms in the BNC and coupled charge transfer at the molecular level is only beginning to emerge [16,21,67]. 

A characteristic feature of the A family COX enzymes is the presence of two proton-conducting pathways (D and K channels) originating at the cytoplasmic side of the membrane and formed by highly conserved proton-exchanging groups [68]. According to the data presented in [7,68,69,70,71,72], these pathways are used to ensure the coupled transfer of protons from the cytoplasmic side of the membrane to the catalytic center (the so-called substrate protons, or protons of the substrate) and to the periplasmic space (the so-called pumped protons, or protons transferred through the membrane). Based on the model formulated in the course of studies of *aa*_3_ oxidases from *P. denitrificans* and *R. sphaeroides*, the K channel serves to transfer some of the substrate protons in the catalytic cycle, while protons pumped through the membrane and the remaining substrate protons are transferred through the D channel. 

The three-dimensional structure of representatives of families B and C is also established (Figure 1) [28,29,30,31]. In the oxidases of the B and C families, only the homolog of the K channel is detected, which presumably serves the transfer of both substrate and pumped protons. The mechanism of proton transfer through this channel in these oxidases remains poorly understood ([73] and references therein). In experiments of different types with family B oxidase, variability in the stoichiometry of proton pumping (0.5–0.85 H^+^/e^−^) is observed. It is assumed that the membrane potential resulting from the operation of the enzyme can lead to a slip of the proton pump at certain stages of the catalytic cycle [27,73]. Theoretically, there is reason to believe that there is a similar decrease in the stoichiometry of proton pumping in the C family oxidase [74]. In addition to the D and K channels, another proposed proton pathway (H channel) was found in heme-copper oxidases of eukaryotes. Based on the structural data on the COX from mitochondria, it is assumed that it can serve for the transfer of protons pumped through the membrane, controlled by redox transformations of low-spin heme (heme *a*) ([58] and references therein). However, the role of the H channel in proton pumping is questioned in experiments with mutant forms on residues in the D, K, and H channels of cytochrome oxidase from lower eukaryotes (yeast) [75]. Thus, in fact, the functional role of the H channel remains unclear. 

The classification of the *bd*-type cytochromes adopted until now is based on the size of the hydrophilic region between transmembrane helices 6 and 7 in subunit I, a binding domain for quinol oxidation designated as the Q-loop. Accordingly, the enzymes are divided into two subfamilies: L (long Q-loop) and S (short Q-loop) [76,77]. Very recently, Murali et al. [4] using phylogenomics identified three families and several subfamilies within the cytochrome *bd* superfamily. According to this classification, all the superfamily members share four transmembrane helices that bind two hemes comprising the catalytic active site. Only one of the three families possesses a conserved quinol binding site. Members of the other two families use cytochrome *c* and possibly another electron donor different than quinol [4]. Notwithstanding, to date all the biochemically characterized *bd*-type cytochromes appeared to be quinol oxidases [14,78,79,80]. Further research is required to obtain and characterize a *bd* enzyme that would use an electron donor other than quinol. 

**Figure 2:**
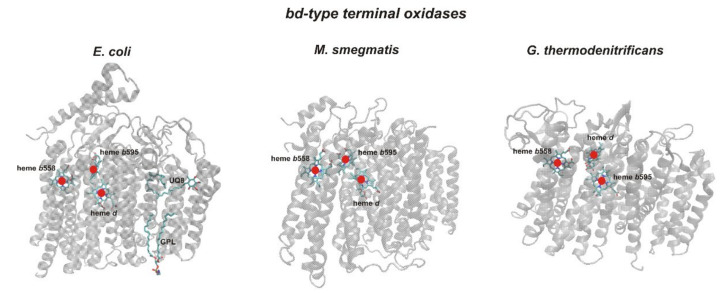
Overall structures of *bd*-type oxidases from *E. coli* ([35], PDB ID: 6RX4), *M. smegmatis* ([81], PDB ID: 7D5I), and *G. thermodenitrificans* K1041 ([33], PDB ID: 5DOQ). The three hemes in all structures are in a triangular arrangement but positions of heme *b*595 and heme *d* in the enzymes from *E. coli* and *M. smegmatis* are interchanged as compared to those in the *G. thermodenitrificans* oxidase. The figure was carried out using Visual Molecular Dynamics software (v1.9.3) [32].

The structures of *bd*-type cytochromes from three different bacterial species determined at 2.7–3.3 Å resolution have been published by now. These are the crystal structure of the *Geobacillus thermodenitrificans* enzyme [33], and the single-particle cryoelectron microscopy (cryo-EM) structures of cytochromes *bd* from *E. coli* [34,35] and *Mycobacterium smegmatis* [81]. The structures show that the enzymes have a similar overall architecture. All structures are characterized by a triangular arrangement of the hemes *b*558, *b*595, and *d*. However, in the oxidases from *E. coli* and *M. smegmatis*, hemes *d* and *b*595 forming the DHAS are found in a ‘switched’ position with respect to the *G. thermodenitrificans* enzyme (Figure 2). Furthermore, the *bd* enzymes from the three bacteria differ in oxygen pathways that they may use. In the *E. coli* oxidase, it is a small hydrophobic channel that allows O_2_ to diffuse from the membrane interior to heme *d* positioned at the center of subunit I (O_2_ channel 1) [34,35]. In the *G. thermodenitrificans* enzyme, heme *d* located closer to the extracellular site is directly connected to the protein surface by an accessible oxygen pathway (O_2_ channel 2) [33]. The *M. smegmatis* cytochrome *bd* may utilize both oxygen pathways, channels 1 and 2, indicating that heme *b*595 located closer to the periplasmic side could be the second O_2_-reduction site [81]. It was also reported that in the respiratory chain of *E. coli*, the *bd* oxidases can be part of supercomplexes [9]. Cytochrome *bd*-I together with formate dehydrogenase and cytochrome *bo* forms a formate:oxygen oxidoreductase supercomplex in a 1:1:1 stoichiometry. In turn, a succinate:oxygen oxidoreductase supercomplex is composed by cytochrome *bd*-II and succinate:ubiquinone oxidoreductase of unknown stoichiometry [9].

Cytochrome *bd* has at least one proton-conducting pathway that connects the cytoplasm to the oxygen reducing center in subunit I [33,34,35,81,82,83]. The structures of cytochromes *bd* from *G. thermodenitrificans* and *M. smegmatis* indicate a second putative proton transfer pathway in subunit II [33,81]. The pathway leads from the cytoplasm to the DHAS. Whether this pathway is functional is not known yet. 

Based on general considerations, the organization of the proton-conducting input pathways in *bd*-type oxidases should be simpler since the major function of the A family HCOs is a generation of the proton motive force whereas the *bd* enzymes do not pump additional protons across the membrane. Instead, cytochromes *bd* play essential roles in bacterial physiology and pathogenesis [14,80,84,85,86,87,88]. The mechanism of transfer of pumped protons along the same pathway (at least a significant part of it) as is used for the transfer of substrate protons (D channel in the A family oxidases; K channel in oxidases of the B and C families) requires a special device to prevent access of pumped protons to the oxygen-reductase center. Although there is no such requirement in the case of *bd*-type oxidases, the pathway for the release of protons leaving the quinol oxidation site must certainly be isolated from the pathways for proton transfer to the oxygen-reductase center of cytochrome *bd*. Only in this case, the *bd* enzyme is able to generate a proton motive force during catalysis, in accordance with the experimental data [82,83,89,90,91]. Indeed, this is confirmed by structural data [33,34,35,81]. It should also be noted that this requirement must also be met in the case of the quinol-binding site of the *bo* oxidase and the pathways for the pumped proton to exit to the outer side of the membrane in heme-copper cytochrome *c* oxidases [29,92].

In accordance with the important physiological role of the *bd*-type oxidases, there is accumulating evidence that cytochromes *bd* make bacteria resistant to nitric oxide (NO) [93,94,95,96,97,98,99,100,101,102], peroxynitrite [85,103], H_2_O_2_ [14,104,105,106,107], cyanide [108,109,110], sulfide [110,111,112,113], and ammonia [114]. The ability of the *bd* oxidases to degrade these hazardous compounds is provided not only by the efficiency of their binding to the reaction center and the transfer of electrons to it, but also by the supply of protons through the proton-conducting input pathways. The features of these side reactions, despite their importance, remain largely unexplored. Since the *bd* oxidases are absent in humans and animals, it seems promising to use them as protein targets for new antibiotics [115,116,117,118,119,120].

## 2. States of Active Sites as Intermediates of the Catalytic Cycle of Oxygen Reduction

**Figure 3:**
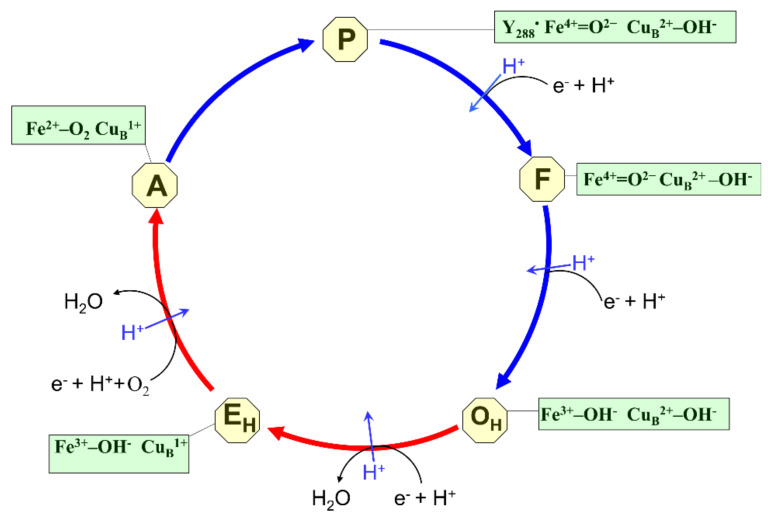
Catalytic cycle of the A family heme-copper oxidases. Shown are catalytic intermediates (O_H_, E_H_, A, P, F), and the structure of the binuclear heme-copper active site for each intermediate. The cycle can be divided into two parts, reductive and oxidative. The reductive part comprises transitions from O_H_ to A. The oxidative part comprises transitions from A to O_H_. Pumped protons are shown by the blue arrows.

The catalytic cycle of COX consists of two half-reactions (reductive and oxidative), each including two single-electron transitions (Figure 3). The reductive phase in the newly oxidized “unrelaxed” enzyme (O_H_ state) involves the sequential transfer of two electrons through the input redox centers to the catalytic center, towards heme *a*_3_ and Cu_B_ (O_H_→E_H_ and E_H_→R transitions, respectively). As a result, the BNC is reduced from the fully oxidized state (O_H_) to the reduced state (R, not shown in Figure 3) and acquires the ability to bind molecular oxygen (see [2,121] and references therein).

The oxidative phase begins with the oxygen molecule binding to the central iron atom of the high-spin heme *a*_3_ in the reduced BNC. As a result, the primary diatomic oxygen adduct is formed (state A), which is a mixture of Fe^2+^–O_2_ and Fe^3+^–O_2_^−^ states. At the next stage, the O–O bond is cleaved, and compound P is formed, for which four electrons and a proton are simultaneously transferred from the active center to the O_2_ molecule. Three electrons result from the oxidation of the Fe^2+^ ion of heme *a*_3_ to the oxoferryl state (Fe^4+^= O^2^^−^) and Cu_B_^+^ oxidation to Cu_B_^2+^. The fourth electron and the proton come from the closely located conserved tyrosine residue (Y288 in the *aa*_3_ oxidase from *R. sphaeroides*) that forms a covalent bond with one of the histidine ligands of Cu_B_ during protein post-translational modification [122,123,124]. 

Two electron vacancies arising in the COX catalytic center are filled by the transfer of the third and fourth electrons from cytochrome *c* molecules. The transfer of the third electron from cytochrome *c* to Y288 leads to the formation of the F state of the BNC in the COX catalytic cycle. The transfer of the fourth electron from cytochrome *c* to heme *a*_3_ reduces the oxoferryl state of heme *a*_3_ into the O_H_ oxidized state. 

**Figure 4:**
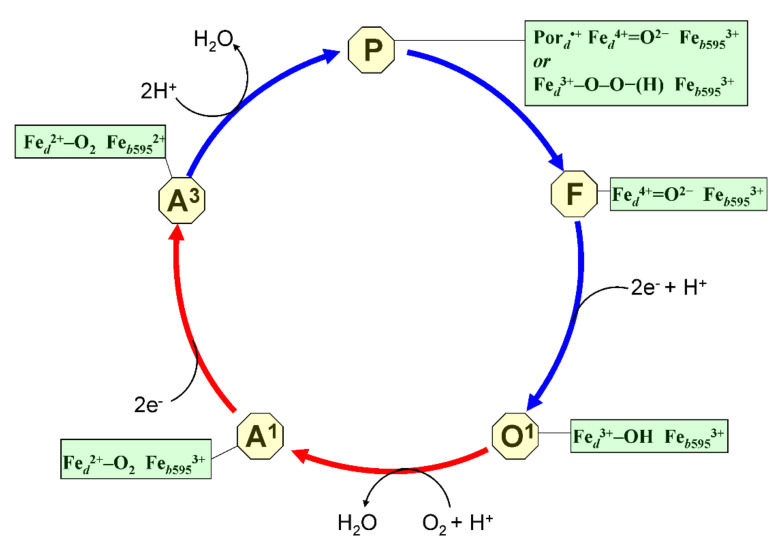
Catalytic cycle of *bd*-type oxidase. Shown are catalytic intermediates (O^1^, A^1^, A^3^, P, F), and the proposed structure of a di-heme active site for each intermediate. The cycle can be divided into two parts, reductive and oxidative. The reductive part comprises transitions from O^1^ to A^3^. The oxidative part comprises transitions from A^3^ to O^1^.

The proposed catalytic cycle of cytochrome *bd* is shown in Figure 4. It can also be divided into two half-reactions, reductive and oxidative. Quinol is involved in the cycle as a two-electron-donating substrate [125]. The reductive part comprises two successive transitions. The first, O^1^→A^1^ transition includes the intramolecular transfer of an electron from the input redox site (heme *b*558) to heme *d* with concomitant binding of O_2_ to the latter heme. Presumably, in this transition, a proton is also transferred to the hydroxide ligand of heme *d* to release a water molecule. The second, A^1^→A^3^ transition involves the transfer of two electrons from the first quinol molecule to hemes *b*558 and *b*595. As a consequence, the fully oxidized DHAS becomes fully reduced with O_2_ bound to heme *d*.

The first reaction of the oxidative part of the cycle is A^3^→P transition [89]. The structure of compound P is controversial. The O–O bond splitting at this stage has either already happened or not yet. In the latter case, P is a true peroxy complex of heme *d* (Fe*_d_*^3+^–O^−^–O^−^– (H^+^)). In the former case, the concerted transfer of four electrons from the DHAS to the bound O_2_ molecule is required. Three electrons come from the ferrous heme *d*. This converts heme *d* (Fe*_d_*^2+^–O_2_) into the oxoferryl state (Fe*_d_*^4+^ = O^2−^) with a porphyrin π-cation radical. The fourth electron is provided by the ferrous heme *b*595 [89,126]. Two protons could also be transferred to the oxygen molecule to produce one more water molecule. If P is a compound I intermediate, the next stage involves the radical quenching by the transfer of an electron from the ferrous heme *b*558 (P→F transition). The oxidative part of the reaction ends with the transfer of two electrons from the second quinol molecule and probably a proton to the DHAS (F→O^1^ transition). This reduces the oxoferryl state of heme *d* into the ferric state with a bound hydroxide ligand. The DHAS becomes completely oxidized whereas the input redox site, heme *b*558, gets reduced. The P→F and F→O^1^ transitions are coupled to membrane potential generation [82,83,91]. The proposed formation of the bound hydroxide ligand and water molecules in the above-mentioned transitions still awaits experimental support.

### 2.1. O/O_H_

In the absence of electron donors, the oxidized “unrelaxed” O_H_ state is spontaneously converted into the oxidized stable state (O) within the second time scale. This process is accelerated at acidic pH and it is believed that the oxidized fast state of COX can only exist at alkaline pH. The O and O_H_ states of the A family cytochrome oxidase differ in their affinity for electrons of the corresponding redox centers and in the ability to perform transmembrane proton transfer [127]. In contrast to the O resting state, injection of the electron into the just oxidized state (O_H_) of the A family *aa*_3_ cytochrome oxidase from *P. denitrificans* which is generated upon oxidation of the fully reduced enzyme by O_2_, results in the rapid electron transfer into the BNC, to Cu_B_. Besides, the electron injection into the O_H_ intermediate of the cytochrome oxidase is coupled to pumping of a proton through the membrane (Figure 3) [127,128]. 

Several experimental investigations on different A family cytochrome *c* oxidases, designed to prepare the O_H_ state, could not find an increase in the Cu_B_(II) midpoint potential as compared to the resting O state, due most likely to the extreme reactivity and short lifetime of the O_H_ state [129,130,131]. At the same time, for the B family cytochrome oxidase *ba*_3_ from *T. thermophilus*, the existence of high and low-energy forms of the oxidized enzyme, which differ in the redox properties of heme centers, is also shown [27,121,127,132,133,134,135].

The 3D structure of the oxidized COX of the A family was solved for the mitochondrial and bacterial enzyme, for the crystals obtained at pH below 7 [59,136]. This structure corresponds to the acidic ‘‘slow’’ form of the enzyme. In this form, Tyr288 is proposed to be protonated. Meanwhile, the ‘‘fast’’ form of the enzyme requires pH 8 or higher. The structural differences between these two states remain to be investigated and are believed to be related to the ligand sphere of Cu_B_ [137]. Recently the structure at pH 7.3 was solved [138]. No structural differences between crystals obtained at the neutral and acidic pH were detected within the molecules. Structure at alkaline pH has not yet been obtained. Recently, the redox sensitivities of the new Raman marker band were observed which suggest that a radical Tyr288 is present in the fast form and a protonated Tyr288 in the slow form [63]. This is consistent with theoretical works [139,140] that in the O_H_ state, the electron is shifted from Tyr to Cu_B_, and the tyrosine is deprotonated in the form of a radical.

### 2.2. O/O^1^

The O^1^ state in cytochrome *bd* was first detected at steady-state in the presence of oxygen and ubiquinol by using stopped-flow spectrophotometry [125]. It was shown that under steady-state conditions, the O^1^ catalytic intermediate is populated up to 20%. Importantly, the O species was reported not to be an intermediate of the *bd* catalytic cycle [141]. The O species can be generated in vitro by incubating the “as isolated” cytochrome *bd* with excess lipophilic oxidant [38,97,114,142]. An apparent difference between O^1^ and O is the presence of an electron on the input redox site, heme *b*558 in O^1^, whereas the DHAS in both states is fully oxidized. Whether there is any difference in redox potentials of the hemes constituting the DHAS between O^1^ and O is not yet known. It is worth mentioning that there is significant redox interaction between heme *b*558 and heme *b*595 [44]. The O^1^ and O states in cytochrome *bd* could be analogs of O_H_ and O in COX.

### 2.3. E/E_H_

The E_H_ state, in contrast to the E state, is high-energy and occurs only during single-electron reduction from O_H_. The single-electron reduction in E_H_ is presumably accompanied by the pumping of a proton through the membrane. Time-resolved experiments showed that there is a difference in the electron redistribution between E and E_H_ states. In the E state, the electron equivalent is distributed among heme *a*, heme *a*_3_ and Cu_B_, whereas in the E_H_ state formed by electron injection into the O_H_ state the electron is located exclusively on Cu_B_ ([134] and references therein).

The redox potential of heme *a*_3_ (the electron acceptor in the E state) is too low (~0.3 V) to provide proton pumping [143]. It is suggested that the E intermediate is formed in an ‘‘activated’’ form E_H_ in which the proton is not on tyrosine but in the center of the BNC forming the second water molecule [144]. Thus, the E_H_ state mixes the low Fe_a3_(III) reduction potential with the higher potential of the tyrosyl radical. The transition from E_H_ to E is presumably accompanied by the transfer of an electron to the tyrosine radical and the protonation of tyrosine. In contrast to O_H_, in the E state, the tyrosine residue is protonated and is no longer in the form of a radical, but in a reduced state [139,140]. In the E_H_ state, the tyrosine residue is either in the form of a radical or it is a mixture of a tyrosine anion and a radical.

In cytochrome *bd*, E/E_H_ (one-electron-reduced) is not a catalytic intermediate. The E state can be produced in vitro by deoxygenation of the “as isolated” cytochrome *bd* under anaerobic conditions, purging the sample repeatedly with argon gas with the aid of the vacuum/gas line [82,145,146]. This turned out to be possible because in the *bd* oxidase isolated under aerobic conditions, the one-electron-reduced A^1^ species predominates [142]. In the oxygen-free E state of cytochrome *bd*, the electron is located predominantly on heme *d*.

### 2.4. R/R^3^

In contrast to the structure of the oxidized cytochrome oxidase, the structure of the reduced enzyme lacks the oxygen ligands of heme *a*_3_ and Cu_B_ [61]. In addition, the hydrogen bond between Tyr288 and the hydroxyethylfarnesyl side chain in the porphyrin ring of heme *a*_3_ disappears, which is believed to open the possibility of proton transfer to the active center through the K channel to break the O–O bond.

R^3^ is not an intermediate of the cytochrome *bd* catalytic cycle. The R^3^ (three-electron-reduced) state can be obtained in vitro by incubating the *bd* enzyme with excess reductant under anaerobic conditions.

### 2.5. A and Ip

Using the CO flow-flash method it was shown that in the A family *bo*_3_ oxidase from *E. coli*, the 42 μs lifetime of the first step (R→A) at 625 μM O_2_ corresponds to a second-order rate constant of ∼3.8 × 10^7^ M^−1^ s^−1^ for O_2_ binding. This rate is ∼2.5 times slower than that observed in the bovine and *R. sphaeroides aa*_3_ enzymes which also belong to the A family [147]. Reduced Cu_B_ is likely to form a transient O_2_ binding site on the way of the oxygen molecule from the membrane via the oxygen channel and into the *a*_3_/Cu_B_ cavity. Transient binding of oxygen to Cu_B_ in the A family oxidases is likely to be weak. It is stronger in the B family oxidases like *ba*_3_ from *T. thermophiles* [26,148]. In contrast to COX, heme *b*595, somewhat analogous to Cu_B_, seems not to be a transient O_2_ binding site on the way of the ligand from outside to heme *d* [41].

The 3D structures of the reduced COX with CO, NO, or CN^-^ respiratory inhibitors, which are competitive inhibitors of O_2_, were used to elucidate characteristics of the A state (the oxy-complex) that is too unstable to be crystallized [21]. The X-ray structures of NO and CO derivatives provided a structural basis for the bend end-on binding of O_2_ to Fe of heme *a*_3_ and the structure of the A state as a mixture of Fe^2+^–O_2_ and Fe^3+^–O_2_^–^ states. This strongly suggests the remarkable stability of the A intermediate and extremely low availability of the second electron equivalent to the bound O_2_ from Cu_B_. On the opposite, the X-ray structure of the fully reduced cyanide anion bound form indicates that cyanide binding significantly influences the Cu_B_ coordination structure. This structure suggests that Fe^3+^–O_2_^−^ induces coordination in the BNC site to form three possible electron-transfer pathways, each transferring one electron equivalent, for the nonsequential three electron reduction in the bound O_2_. These pathways are as follows: from Fe of heme *a*_3_; from Cu_B_; and from Y288-OH via a water molecule (W510). W510 is not a product of the enzymatic reaction but a cofactor stored in a water storage site near the O_2_-reduction site [21,149].

This X-ray structural analysis confirmed earlier theoretical work [150] which suggested that the O–O bond cleavage proceeds through the two-step mechanism. It is initiated by proton transfer from the cross-linked tyrosine, via one or two water molecules (W510 in the structure) to the superoxide, to form the type of peroxide intermediate (Ip), Fe_a3_(III)OOH^–^ Cu_B_(II). The proton transfer is coupled to an electron transfer from the Cu_B_-tyrosine complex. In the second step, the O–O bond is cleaved and the P_M_ intermediate is formed, with Fe_a3_(IV) = O^2−^ plus Cu_B_(II)OH^–^ TyrO* [151,152,153]. Since both an electron and a proton move from the tyrosine in the same direction and distance to the oxygen atom, the A→P_M_ transition does not generate a transmembrane voltage and virtually no phase of potential generation during R→A→P_M_ steps was obtained using the electrometric technique [154] (Figure 3).

The Ip intermediate was experimentally resolved in a recent study on the B family cytochrome oxidase (*ba*_3_ from *T. thermophilus*) at low temperature and pH. Electron transfer from the low-spin heme *b* to the catalytic site was shown to be faster by a factor of ~10 (τ ~ 11 μs) than the formation of the P_R_ ferryl (τ ~ 110 μs). This indicates that O_2_ is reduced before the splitting of the O–O bond [152].

The significantly faster O_2_ binding and the O–O bond cleavage in the *T. thermophilus ba*_3_ as compared to analogous steps in the *aa*_3_ oxidases could reflect evolutionary adaptation of the *ba*_3_ enzyme to the microaerobic conditions of the *T. thermophilus* HB8 species [155]. In evolutionarily distant *bd* oxidases, nevertheless, the same problem is solved-survival at low oxygen levels.

### 2.6. A^1^ and A^3^

Unlike COX, the presence of a single electron in the DHAS of cytochrome *bd* is sufficient to form a stable globin-like oxy-complex. The likely reason for this is the high oxygen affinity of ferrous heme *d*, at least in *E. coli* and *Azotobacter vinelandii* [145,146]. Significant amounts of A^1^ are observed in the *bd*-containing bacterial membranes and the isolated enzyme, as evidenced by an absorption band near 650 nm typical of the heme *d* oxy-complex [142,156,157]. Stopped-flow experiments showed that at steady-state about 40% of cytochrome *bd* is in the A^1^ form [125].

Transient formation of A^3^ was registered by the flow-flash method with a microsecond time resolution [89,146,158]. The fully reduced CO-bound cytochrome *bd* was photolyzed in the presence of O_2_. The flash-induced absorption changes associated with the reaction of oxygen with the oxidase were detected. The rate of the A^3^ production depends linearly on the O_2_ concentration giving a second-order rate constant *k*_on_ of ~2 × 10^9^ M^−1^s^−1^. The formation of A^3^ from the fully reduced enzyme is not electrogenic [82,83,89,91].

The available structures show that the *bd* enzymes from *G. thermodenitrificans* and *E. coli* use different pathways for oxygen delivery [33,34,35]. This is likely due to the fact that the O_2_-binding site, heme *d*, is located closer to the periplasmic side in the *G. thermodenitrificans* structure, whereas in the *E. coli* structure it is positioned approximately in the middle of the membrane. As a result, in the former structure, O_2_ can get access to heme *d* via an oxygen entry site located laterally at a short distance from the heme. There is no need for a functionally conserved protein cavity for the gaseous substrate to bind to heme *d* [33]. In the latter structure, the oxygen pathway is parallel to the membrane plane and connects the lipid interface to heme *d* [34,35]. It is provided by a specific hydrophobic channel that starts near Trp63 of CydB and extends towards heme *d* on CydA.

### 2.7. P and F

Similar to peroxidases, the reduction of the state P (analog of compound I) by two electrons through the intermediate state F (analog of compound II) converts the enzyme into the oxidized state O_H_ (third and fourth electrons in the catalytic cycle). The P and F states of cytochrome oxidase contain heme iron *a*_3_ in the ferryl-oxo state. An essentially identical A 1.70 Å Fe–O distance of the ferryl center of heme *a*_3_ which was resolved in the X-ray structures of catalytic intermediates (P and F) could best be described as Fe*_a_*_3_^4+^ = O^2−^ rather than Fe*_a_*_3_^4+^–OH^−^ [57]. In that work they found an interstitial water molecule that could trigger a rapid proton-coupled electron transfer from tyrosine-OH to the slowly forming Fe*_a_*_3_^3+^–O^−^–O^−^–Cu_B_^2+^ state preventing its detection, consistent with the unexpected Raman results.

The state P differs from F by an additional oxidative vacancy located on the aromatic residue near the binuclear center, covalently bound to one of the His ligands of Cu_B_. This aromatic residue, tyrosine Y288, is suggested to provide the fourth electron during the cleavage of the O–O bond. The tyrosine radical Y288 is able to exchange with other aromatic residues (for example, W236), but at what rate this occurs and which radical is the primary one is not fully understood [152].

Flow-flash method in combination with electrometric or spectroscopy technics allows us to follow the charge transfer steps during the catalytic cycle of the proton-motive force generators [159,160,161,162,163]. In the case of the *caa*_3_ oxidase from *T. thermophilus* which has five electron equivalents in the reduced state, the electrogenic component with τ ~ 50 μs is associated with the P_R_→F transition. Together with the previous reaction step (A→P_R_) this is coupled to the translocation of about two charges across the membrane. The three subsequent electrogenic phases, with time constants of ~0.25 ms, ~1.4 ms, and ~4 ms, are linked to the conversion of the binuclear center through the F→O_H_→E_H_ transitions, and result in the additional transfer of four charges through the membrane dielectric [134,135].

Earlier, using electrometric techniques and direct pH measurements of the A family *aa*_3_ oxidases, it was also shown that the formation of the F state from the P state and the F to O_H_ transition are coupled to the pumping of protons across the membrane [164,165,166,167,168,169,170] (Figure 3). Each of these transitions is associated with the absorption of two protons from the internal solution of proteoliposomes with incorporated cytochrome *c* oxidase and the release of one proton into the external bulk phase. The E_H_→R transition is the least explored with a time resolution and consequently less understood step of the catalytic cycle due to technical difficulties with stabilization of cytochrome *c* oxidase in this state. Assuming that the O_H_→E_H_, F→O, and P_M_→F transitions are coupled to the pumping of approximately one proton, the resting forth pumped proton should be associated with the E_H_→R transition [171]. Thus, the experiments performed with a microsecond time resolution directly indicate that each of the four one-electron transitions in the catalytic cycle of the A family cytochrome *c* oxidase (highly effective generator of a proton motive force) is coupled to the pumping of one proton through the membrane (Figure 3) [69,70,134,135,166,171,172,173,174]. At the same time, in the B family oxidases, some time-resolved one-electron transitions (O_H_→E_H_ [27] and P→F [26] or F→O, depending on the interpretation [19]) are not associated with the transfer of the pumped proton that explains the decrease in pumping stoichiometry in these oxidases (~0.5 H^+^/e vs. 1 H^+^/e) observed under certain conditions in stationary measurements.

In cytochrome *bd*, P was discovered as a short-lived catalytic intermediate in-between A^3^ and F in the flow-flash kinetic experiments [89]. It is formed from A^3^ nonelectrogenically with τ of 4–5 μs at 21 °C [89,90]. The chemical structure of P is still a matter of debate. In the original paper, Belevich et al. [89] suggested that P is either a true peroxy complex (analog of compound 0) or oxoferryl species with a radical on the porphyrin ring or an amino acid residue (analog of compound I). The authors leaned towards the former possibility because the radical formation would be energetically unfavorable in the presence of the reduced heme *b*558 in the proximity of the DHAS. Later, Paulus et al. using ultra-fast freeze-quench trapping followed by EPR and absorption spectroscopy reported that P is a heme *d* oxoferryl porphyrin π-cation radical intermediate that magnetically interacts with heme *b*595 [126]. It has to be noted that Paulus et al. performed the experiments at non-physiological, lower temperatures. It cannot be excluded that “P” observed by Belevich et al. is a mixture of the peroxy and oxoferryl cation radical species provided that they have similar spectral characteristics. Whatever the P intermediate is, at the next catalytic step it decays into a nonradical F species (analog of compound II), concomitant with the oxidation of heme *b*558. This transition occurs with τ of 47 μs (at 21 °C) and is coupled to the membrane potential generation [89,90]. The F species absorbing maximally at 680 nm is persistently seen in the static spectrum of the “as-prepared” cytochrome *bd* and was first identified as oxoferryl (Fe*_d_*^4+^=O^2−^) by resonance Raman spectroscopy [175]. At steady-state the F species is populated up to 40% [125].

## 3. Noncatalytic States of Active Sites That Occur When External Ligands Are Added: Heme-Copper, *bd*

Due to the fact that the active centers of terminal oxidases are able to interact with a wide variety of external ligands, we consider only a few examples illustrating this variety.

The states of the binuclear center, similar to the states P and F, can also be obtained in the reaction of an oxidized cytochrome oxidase of the A family with H_2_O_2_. It is possible to solve the kinetics of the formation of intermediates P and F in the reaction with peroxide using the fast mixing method. For example, the first intermediate of *caa*_3_ cytochrome oxidase from *T. thermophilus* is formed with a bimolecular constant of 1540 M^−1^s^−1^ and has characteristics in the visible region and Soret band similar to the P intermediate resolved in the kinetics of enzyme oxidation in the single-turnover mode [134]. The second intermediate is formed with a constant of 21.5 M^−1^s^−1^ and has spectral characteristics similar to the intermediate F. The addition of the second molecule of H_2_O_2_ converts P to the F state. It is suggested that in presence of peroxide, the P-to-F transition is due to the binding of H_2_O_2_ to Cu_B_ triggering a structural change together with the uptake of H^+^ at the catalytic center, complemented with the annihilation of Y288 radical by the intrinsic oxidation of the enzyme [176].

In contrast to the A family oxidases, the binding of peroxide to the B family oxidases in a fully oxidized state is not observed in similar experiments. It is assumed that the BNC is in a closed state and peroxide begins to bind only when the enzyme is reduced by a single electron [132].

**Figure 5:**
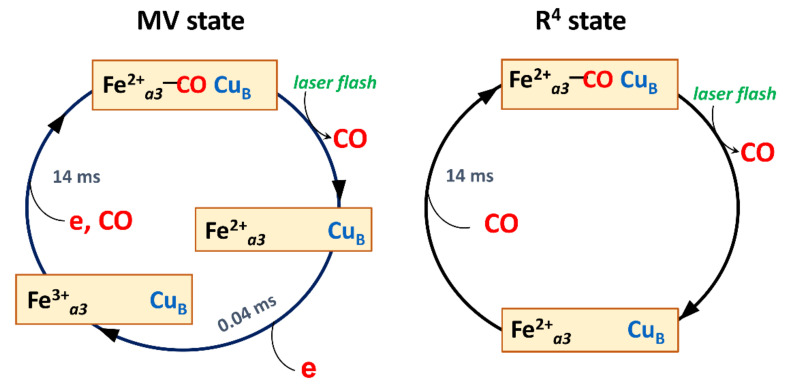
Proposed scheme for a cycle of photolysis and recombination of CO to the heme-copper cytochrome oxidase *caa_3_* from *T. thermophilus* (A2 family) in the two-electron-reduced mixed-valence (MV) and fully reduced (R^4^) states. In the R^4^ state, photodissociation of CO from the ferrous heme *a_3_* iron (Fe^2+^*_a_*_3_) is followed by its rebinding to the heme with τ of 14 ms. In the MV state, in addition to the CO rebinding, there is a transient back-flow of an electron from the binuclear center to the input redox center (heme *a*, Cu_A_, and cytochrome *c*) with τ of 0.04 ms. Intermediate binding of CO to Cu_B_ is not shown. The scheme is based on time-resolved spectroscopic studies [26,135].

The interaction of the reduced BNC with CO in heme-copper oxidases allows us to study the kinetics of the ligand migration to the binuclear center (CO recombination) and the stages of the reverse electron transfer caused by photolysis of the bond of CO with the iron atom of a high-spin heme by a short laser flash. As typical for the heme-copper oxidases, upon photodissociation of CO from the mixed-valence *caa*_3_ cytochrome oxidase from *T. thermophilus* in the absence of oxygen the reverse electron transfer from heme *a*_3_ to heme *a* and further to the cytochrome *c*/Cu_A_ pair is resolved as a single transition with τ ~ 40 μs. Unlike the mitochondrial cytochrome oxidase and typical prokaryotic oxidases with four redox centers, this enzyme has an additional redox center, cytochrome *c*. Electron exchange between Cu_A_ and the additional cytochrome *c* in this enzyme proceeds much faster, therefore it is not isolated as a separate kinetic stage. The electron back-flow is followed by the return of an electron to the BNC and rebinding of CO with a rate constant of ~14 ms, a process that is only observed in the fully reduced state (Figure 5). Unlike the A family oxidases in which there is a significant reverse electron transfer from the BNC to the input redox centers, in the B family oxidases, this transfer is much smaller and does not exceed a few percentage points [26,155]. This is likely due to the lower redox potential of the low-spin heme of the B family oxidases as compared to the BNC under these conditions. Similarly, in the *bd* oxidases, the reverse electron transfer is also not significant under these conditions [45,47,48,82,177].

When ammonia is added at pH 9 to the F state, classically generated by reaction with excess H_2_O_2_, a new P_N_ state (in contradiction to the conventional direction of the catalytic cycle) is shown to appear. It is suggested that ammonia coordinates directly to Cu_B_ in the binuclear active center in this P state [178]. The structural and functional properties of this artificial intermediate compound require further research.

A species with an absorption maximum at 680 nm can also be produced by the addition of excess H_2_O_2_ to the *bd* oxidase being in the O or “as-prepared” state [82,94,179,180]. As its spectral features are similar to those observed for the F intermediate in the flow-flash and stopped-flow experiments [82,89,90,125], it can be suggested that these species also have similar or even identical chemical structures. In other words, both are likely a heme *d* oxoferryl intermediate.

The oxygen-binding site of cytochrome *bd*, heme *d*, can also bind external ligands. Usually, a ferrous heme binds electroneutral molecules whereas a ferric heme binds ligands in the anionic form. In the case of heme *d*, it is true for cyanide and CO but not for NO.

MCD and absorption spectroscopy shows that cyanide binds to ferric heme *d* in the O state of cytochrome *bd* [38]. At a high concentration, the ligand can also react with part of ferric heme *b*558 in the isolated enzyme [38,181]. However, if the enzyme is then reconstituted into phospholipid-based liposomes, cyanide no longer interacts with heme *b*558 and binds predominantly to the heme *d* site [181]. Interestingly, the MCD study does not reveal any substantial cyanide binding to the high-spin pentacoordinate ferric heme *b*595, even at the highest concentration of the ligand used, 50 mM [38]. According to the EPR study, conversely, the addition of the ligand to cytochrome *bd* results in its binding to the DHAS as a bridging ligand (Fe*_d_*^3+^–C = N–Fe*_b__595_*^3+^) [37]. As discussed below, it is quite unlikely. The Fe–Fe distance between hemes *d* and *b*595 is too big to allow a bridging structure in the DHAS. Resonance Raman studies report that in the cyanide complex heme *d* is high-spin pentacoordinate [182,183]. This suggests that the binding of cyanide to heme *d* is accompanied by dissociation of the heme axial ligand.

The addition of CO to cytochrome *bd* in the fully reduced state (R^3^) leads to the ligand binding to ferrous heme *d* [38,40,49,77,184,185] with a high affinity [43,186]. The formation of heme *d*-CO adduct is accompanied by a small spectral perturbation of heme *b*595 [39,41,45]. The binding of the ligand with ferrous heme *b*595 as such is usually minor. In the case of the *bd* oxidases from *E. coli* and *A. vinelandii*, the CO-reactive fraction of heme *b*595 does not exceed 5–15% under different experimental conditions [36,40,46]. However, in the *bd* enzyme from *G. thermodenitrificans*, this fraction is larger, 20–25% [77].

**Figure 6:**
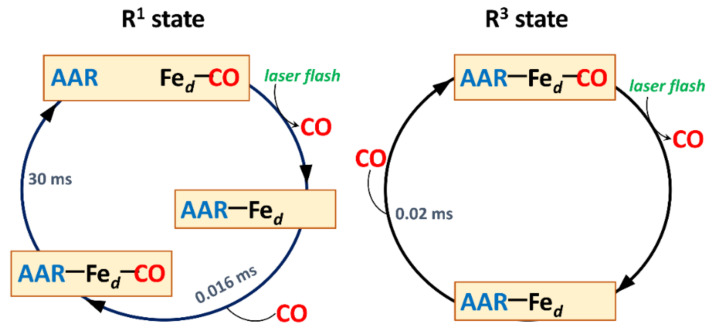
Proposed scheme for a cycle of photolysis and recombination of CO to *bd*-type oxidase in one-electron-reduced (R^1^) and fully reduced (R^3^) states. In the R^1^ state, photodissociation of CO from the ferrous heme *d* iron (Fe*_d_*) is accompanied by binding of the axial ligand, an amino acid residue (AAR), at the opposite side of the heme. CO rebinds to Fe*_d_* with τ of 0.016 ms producing a transient hexacoordinate state (AAR–Fe*_d_*–CO). Then AAR is dissociated from Fe*_d_* with τ of 30 ms. In the R^3^ state, AAR is a permanent indissociable ligand to Fe*_d_*. Photodissociation of CO from Fe*_d_* is followed by its rebinding to the heme with τ of 0.02 ms. AAR is either H19 [34] or E99 [35]. The scheme is based on time-resolved spectroscopic studies [47,48,49]. The electron transfer in the opposite direction (back-flow) observed in the R^1^ state occurs to a much lesser extent than in heme-copper oxidases and therefore is not shown in the scheme.

O_2_ in the A^1^ state can be replaced with CO that results in CO binding to ferrous heme *d* and the formation of the one-electron-reduced CO-bound species (R^1^-CO). Studies of photolysis and subsequent recombination of CO with heme *d* showed that the flash-induced dynamics of the heme iron coordination sphere between the R^1^ and R^3^ states of the enzyme is different (Figure 6) [47,48,49]. In the R^3^ cytochrome *bd*, the heme *d*-CO complex is hexacoordinate and the protein-derived axial ligand to the heme iron (amino acid residue, AAR) remains permanently indissociable during photolysis and recombination processes. In the R^1^ cytochrome *bd*, on the contrary, the heme *d*-CO complex is pentacoordinate (i.e., AAR is not bound to Fe*_d_*^2+^) but photodissociation of CO from Fe*_d_*^2+^ is accompanied by transient binding of AAR at the opposite side of heme *d*. AAR is either H19 [34] or E99 [35]. This flexibility of the heme *d* coordination sphere may have functional significance during the catalytic cycle.

Unlike cyanide and CO, NO can react to different states of heme *d*. The reaction of cytochrome *bd* in the A^1^ (Fe*_d_*^2+^–O_2_), R^1^ or R^3^ (Fe*_d_*^2+^) states yields a nitrosyl ferrous adduct (Fe*_d_*^2+^–NO) [38,93,95]. The O enzyme (Fe*_d_*^3+^) reacts with NO producing a nitrosyl ferric species (Fe*_d_*^3+^–NO or Fe*_d_*^2+^–NO^+^) [97]. Reaction between cytochrome *bd* in the F state (Fe*_d_*^4+^ = O^2−^) and NO gives a nitrite-ferric (Fe*_d_*^3+^–NO_2_^−^) derivative [94].

Cytochrome *bd* in the O state also interacts with ammonia. Surprisingly, in contrast to cyanide, CO, and NO, NH_3_ does not inhibit but activates the *bd* oxidase at alkaline pH [114]. It can be hypothesized that NH_3_ promotes the conversion of O into P thereby accelerating the enzyme activity. It is also possible that ammonia reacts with O^1^ yielding F. In these two reactions, NH_3_ may be oxidized to NH_2_OH.

## 4. BNC vs. DHAS

A very short distance between Fe of heme *a*_3_ and Cu_B_ (4.5–5.2 Å, see references in [7]) allows the BNC of COX to bind a single diatomic molecule or ion, e.g., a peroxo group, as a bridging ligand. The Fe–Fe distance between heme *d* and heme *b*595 is much larger (11.1–11.6 Å [33,34,35]) that excludes such a possibility for the DHAS of cytochrome *bd*. Therefore, in this sense, heme *b*595 is not a functional analog of Cu_B_. Nonetheless, the distance between the edges of heme *d* and heme *b*595 is rather small (3.5–3.8 Å [33,34,35]) suggesting they are in van der Waals contacts. The van der Waals interactions enable fast electron transfer between the hemes. The first evidence of this was the observation that the 4.5-μs transition of A^3^ to P is accompanied by the oxidation of heme *b*595 [89]. Later, a CO photolysis/recombination study of the one-electron reduced cytochrome *bd* provided evidence for electron transfer between hemes *d* and *b*595 within 0.2–1.5 μs [47,48]. Thus, heme *b*595 rapidly donates an electron and possibly a proton to heme *d* for the oxygen chemistry occurring in the DHAS. This function of heme *b*595 in cytochrome *bd* is similar to that of Cu_B_ in COX.

Major recent advances in the heme-copper oxidases include the role of the unique tyrosine residue which is now, in addition to the usual Cu_B_ and the iron atom of heme *a*_3_, accepted to be included in the active center. Thus, the active site of cytochrome *c* oxidase comprises an oxygen-binding heme, a nearby copper ion (Cu_B_), and a tyrosine residue that is covalently linked to one of the histidine ligands of Cu_B_ and conserved throughout the superfamily of the respiratory heme-copper oxidases. The most recent results suggest that the catalytic importance of this residue considerably exceeds the original idea of assistance in the breaking of the O−O bond and that it is of key importance in modulating the redox potentials of the catalytic site intermediates and gating of the proton transfer through the K-channel. These redox potentials are now established with reasonable accuracy from both experiments and calculations, and the remarkable “leveling effect” by the active site, relative to the potentials of O_2_ reduction in solution, is more dramatic than originally estimated [16,144]. Hence, the neutral radical form of Tyr288 is suggested to have a more general role in the cytochrome *c* oxidase mechanism than thought previously.

The fact is that in the organization of the active center of heme-copper oxidases, the most important task of thermodynamic alignment (“leveling”) of all four single-electron transitions is solved, providing the necessary amount of free energy for pumping a proton in each single-electron transition when oxygen is reduced to water. At the same time, there is no such tough task for the active center of the *bd* oxidases. There are no indications of the presence of an aromatic radical in the active center or its participation in the catalytic cycle of a *bd* oxidase; when the O–O bond is broken, an additional electron is taken from the heme porphyrin. However, the organization of the *bd* active center can and should meet other requirements that ensure the performance of special physiological and adaptive functions of this unique cytochrome in prokaryotes. More specifically, fine-tuning of the thermodynamics of redox transitions in the active center of the *bd* oxidase can provide the solution of such problems as a high rate of partial stages of oxygen reduction which ensure the adaptive value of these oxidases under oxygen-limited conditions; acceleration or, vice versa, prohibition of side reactions with stress-induced metabolites (inhibitors) ensuring adaptive benefits. The complex structural and functional aspects of these issues for the *bd* oxidases are still under development, for which the necessary prerequisites have now appeared due to the deciphering of the three-dimensional structure of all representatives of the terminal oxidases, including the *bd*-type cytochromes. This seems to be a promising direction and requires further research, both experimental and theoretical.

## 5. Concluding Remarks

Terminal respiratory oxidases are a broad group of membrane enzymes characterized by a variety of organizations of the main catalytic nodes and evolutionary origins. Despite the similarity of the main catalytic function, the reduction in oxygen to water, these enzymes have important functional differences. On the one hand, heme-copper terminal oxidases have a mechanism of transmembrane proton pumping and are therefore more important as effective generators of a proton motive force. The *bd*-type cytochromes and some heme-copper oxidases of minor families, although being less effective energy converters, at the same time provide microorganisms with adaptation to low-oxygen conditions and survival in chemically aggressive environments and, as a result, resistance to antibiotics. Currently, three-dimensional structures of members of all major groups of terminal oxidases, the main families of heme-copper oxidases and *bd*-type cytochromes, have been solved. This provides a unique opportunity for a comprehensive structural and functional study of these important enzymes. Elucidation of the general principles and specific features of the organization and mechanisms of functioning of terminal oxidases of different types will help to decipher the mechanisms of energy conversion in biological membranes and create artificial and hybrid nanoscale objects with desired properties, establish mechanisms for the regulation and adaptation of cellular respiration, the resistance of pathogenic microorganisms, and search for new drugs.
